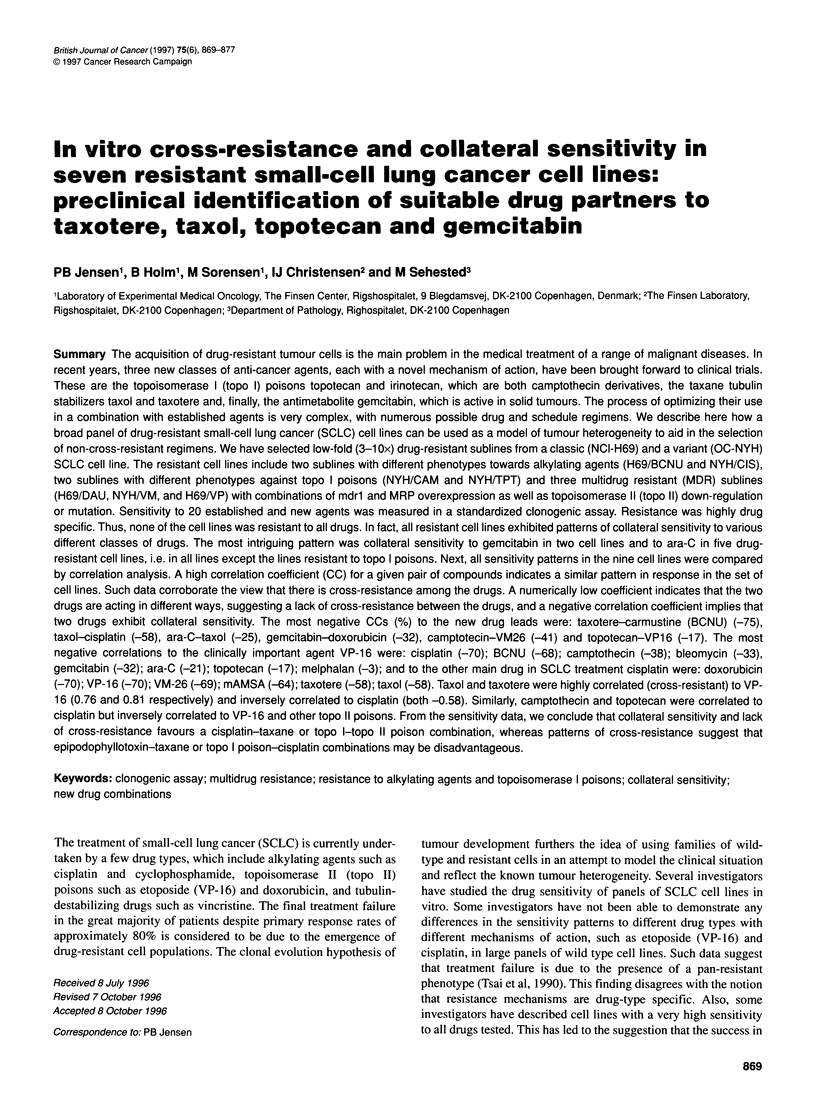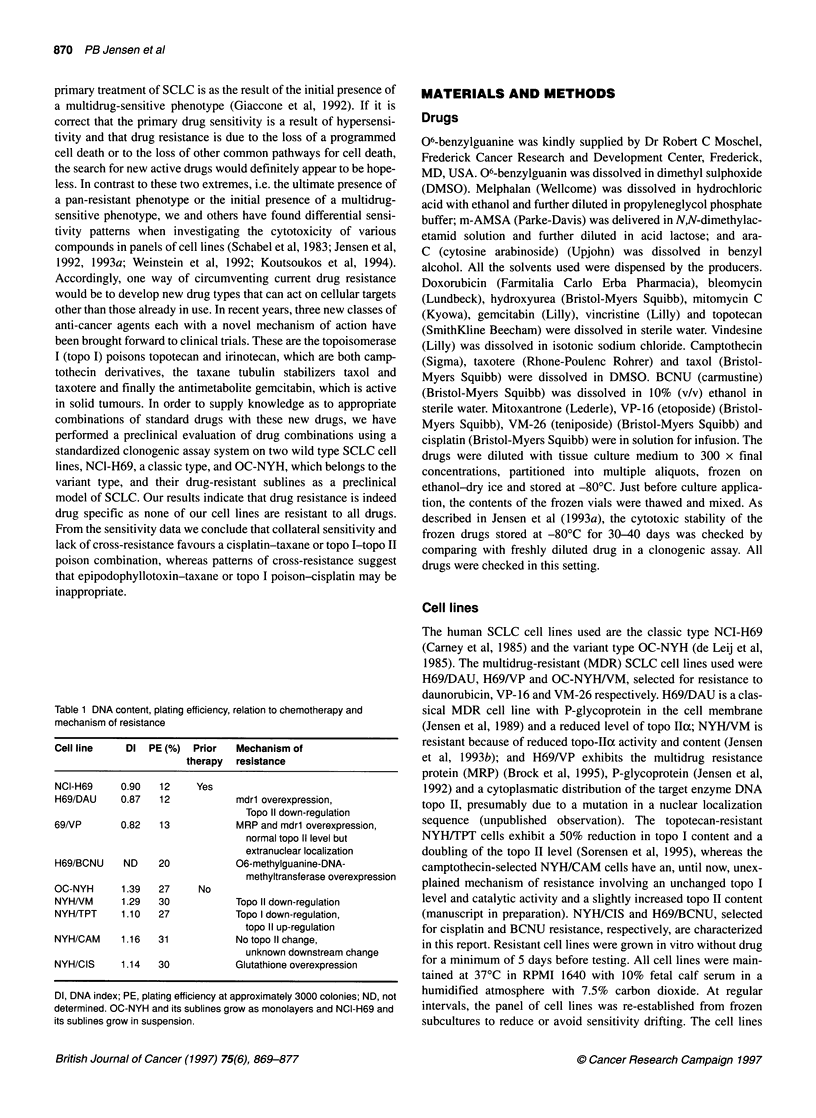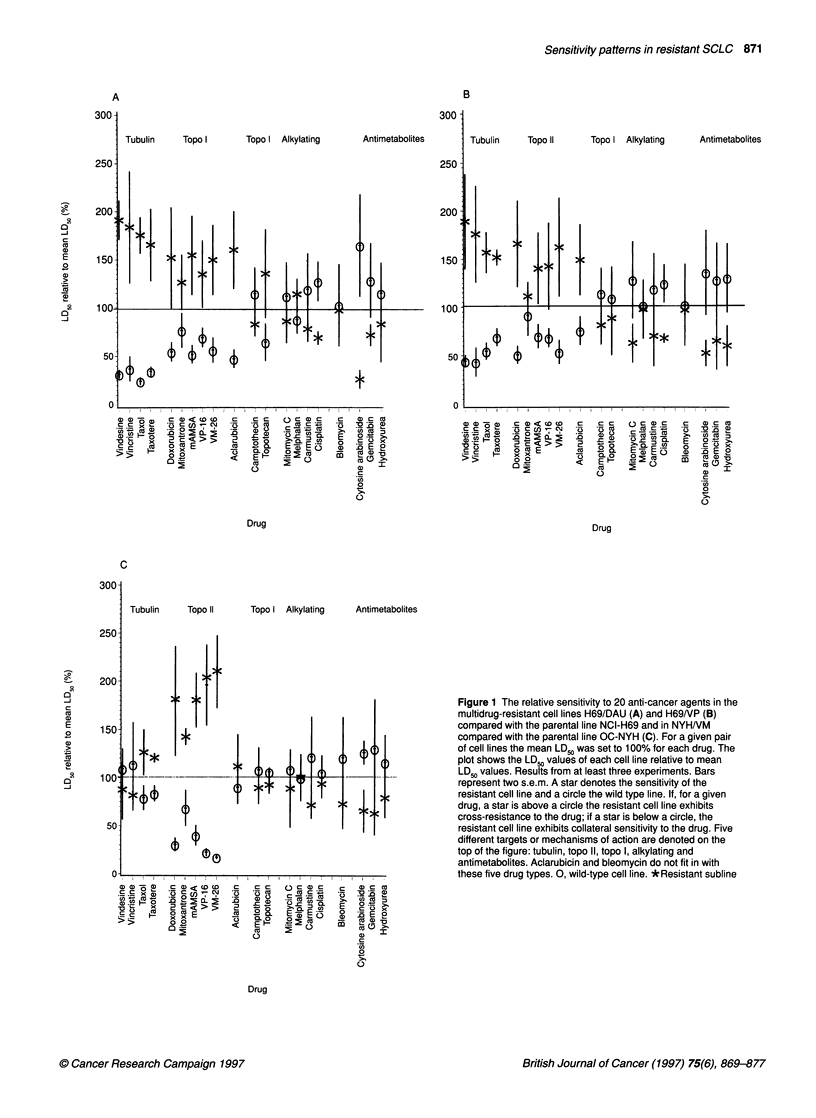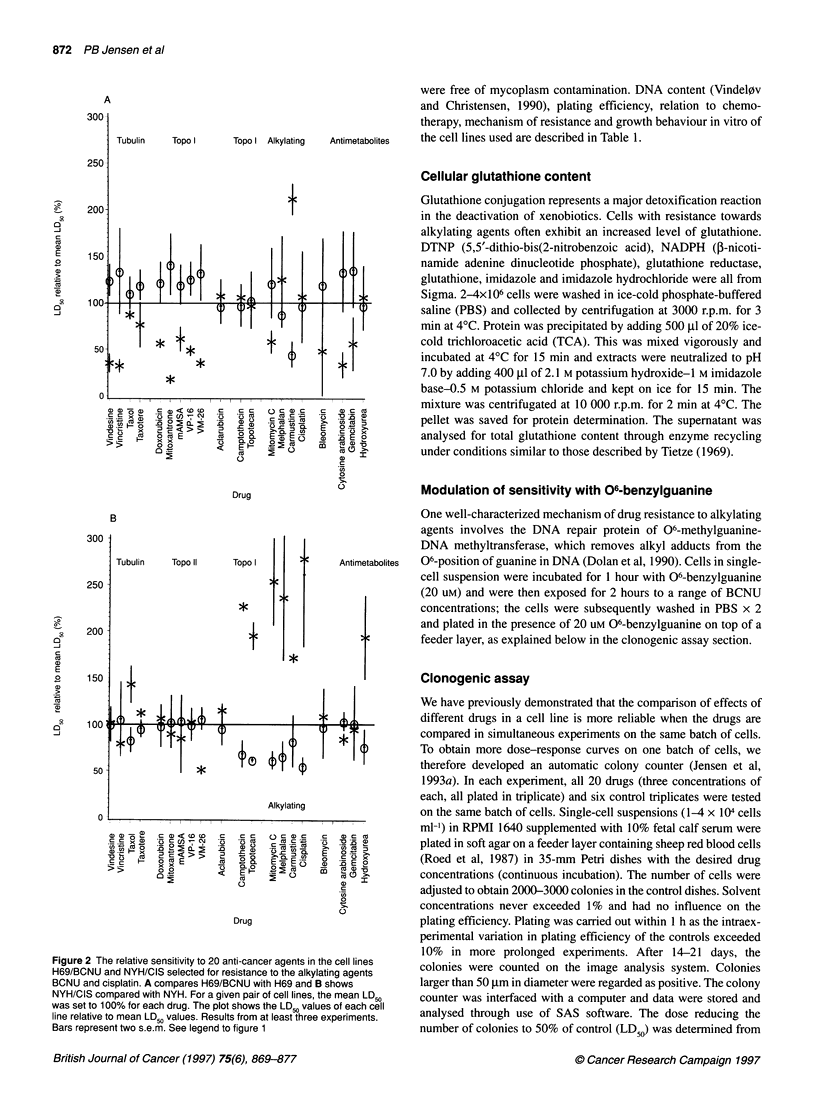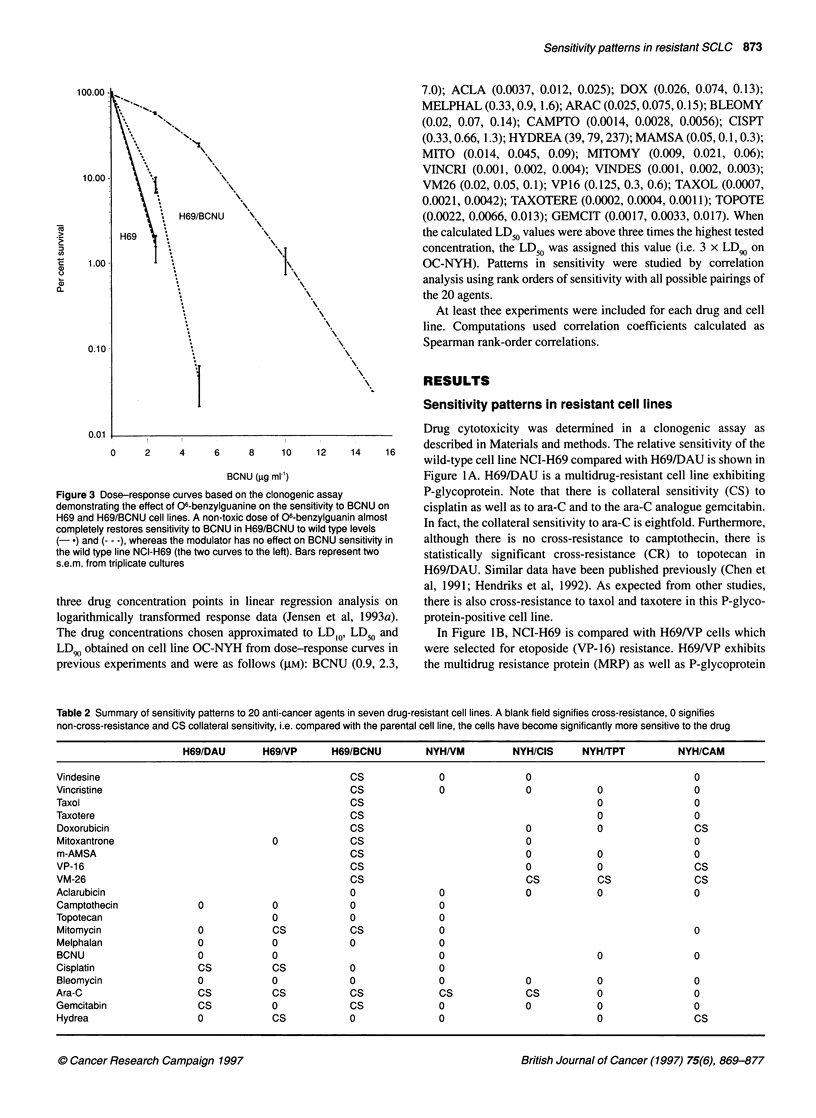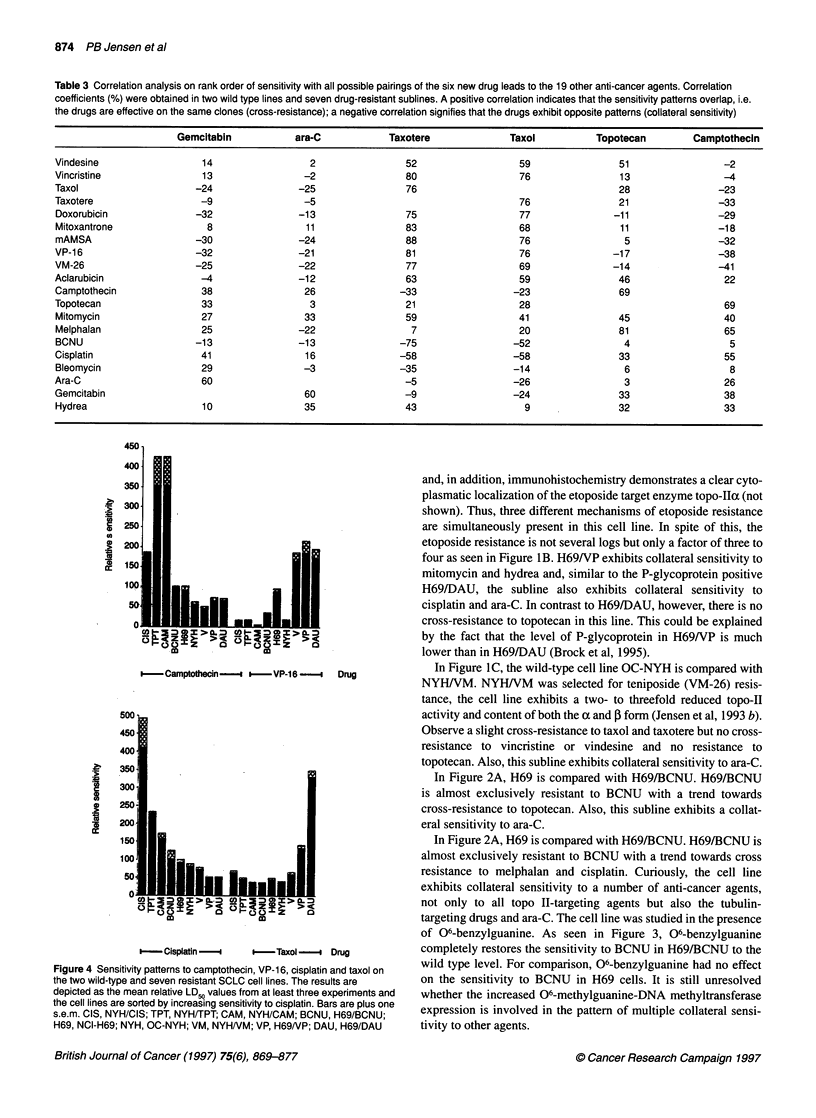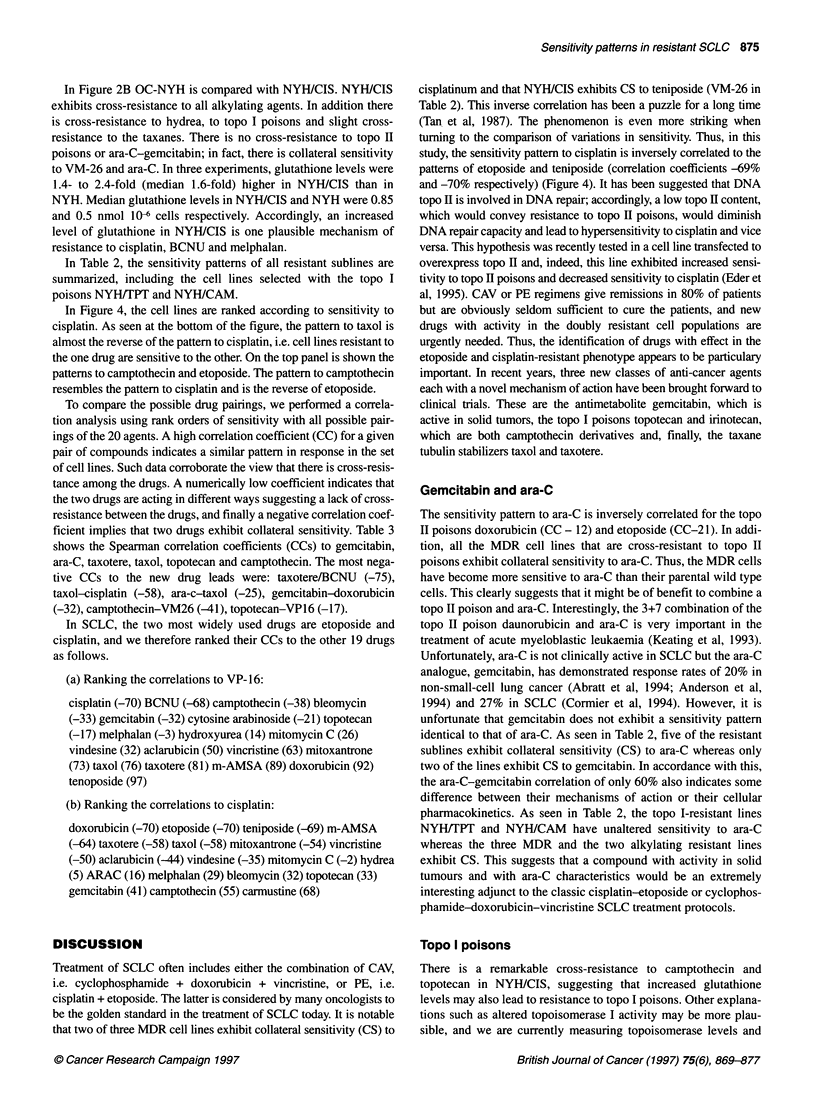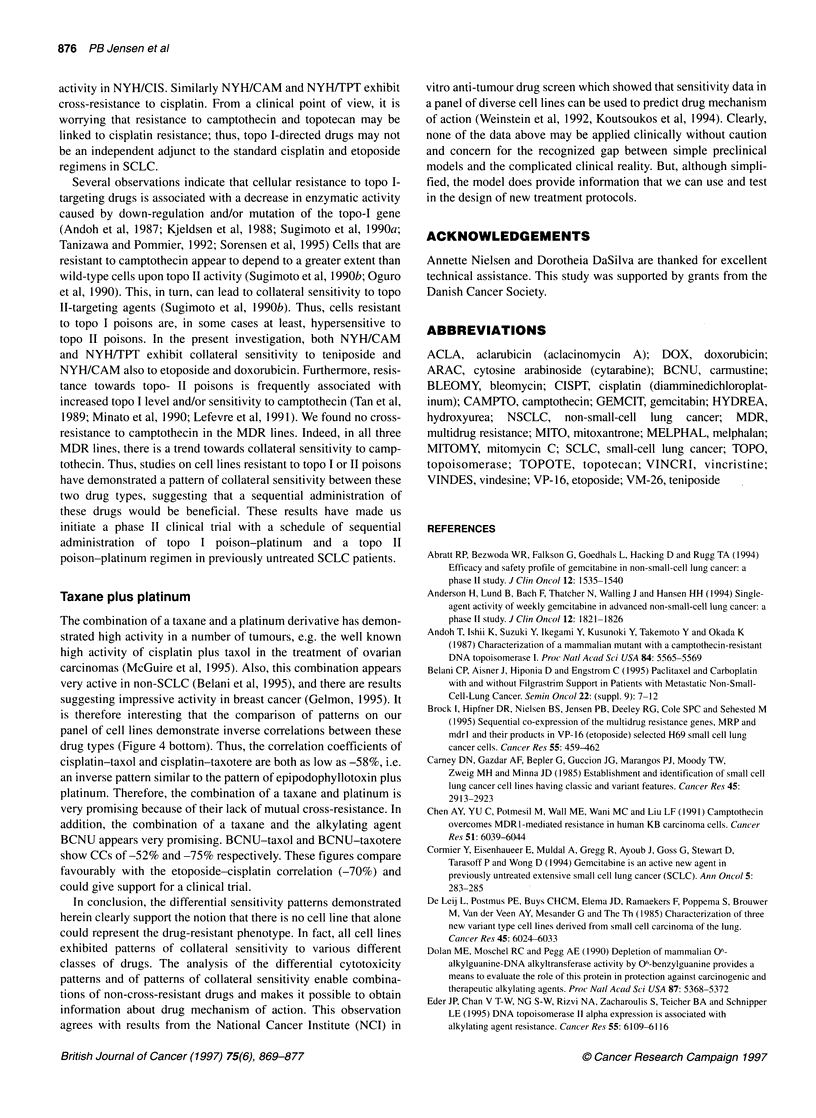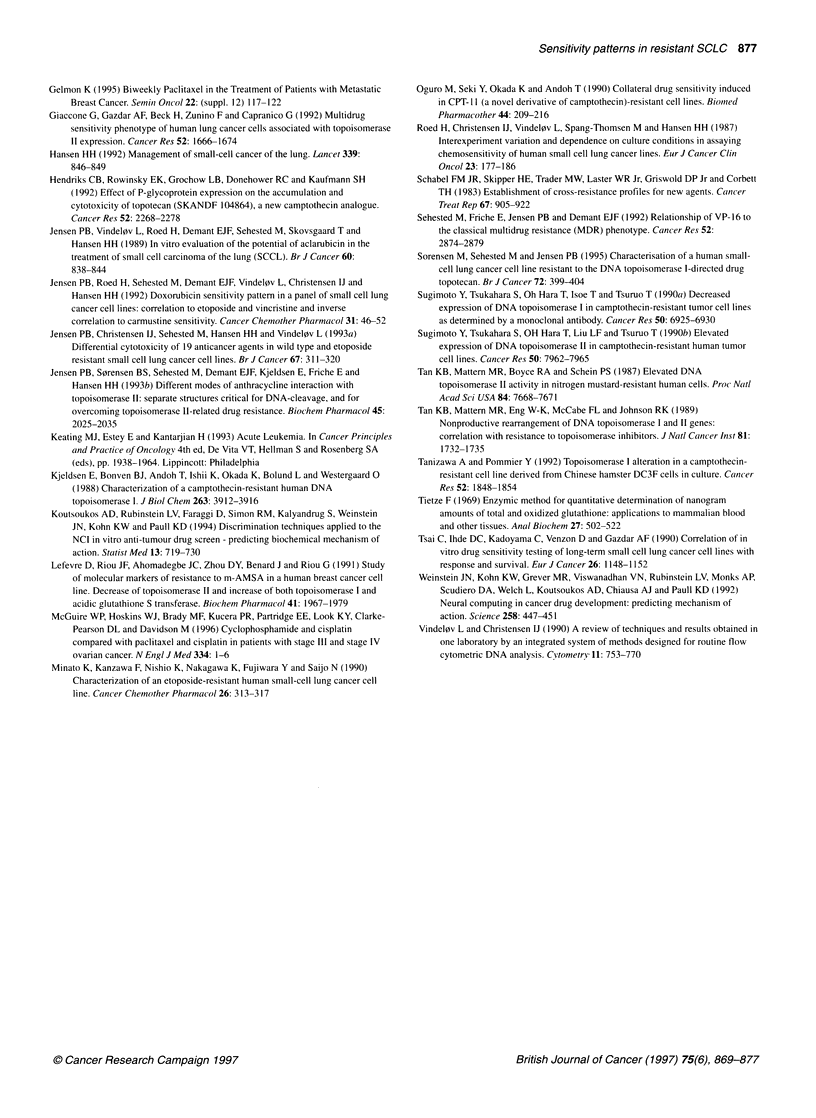# In vitro cross-resistance and collateral sensitivity in seven resistant small-cell lung cancer cell lines: preclinical identification of suitable drug partners to taxotere, taxol, topotecan and gemcitabin.

**DOI:** 10.1038/bjc.1997.154

**Published:** 1997

**Authors:** P. B. Jensen, B. Holm, M. Sorensen, I. J. Christensen, M. Sehested

**Affiliations:** Laboratory of Experimental Medical Oncology, The Finsen Center, Rigshospitalet, Copenhagen, Denmark.

## Abstract

The acquisition of drug-resistant tumour cells is the main problem in the medical treatment of a range of malignant diseases. In recent years, three new classes of anti-cancer agents, each with a novel mechanism of action, have been brought forward to clinical trials. These are the topoisomerase I (topo I) poisons topotecan and irinotecan, which are both camptothecin derivatives, the taxane tubulin stabilizers taxol and taxotere and, finally, the antimetabolite gemcitabin, which is active in solid tumours. The process of optimizing their use in a combination with established agents is very complex, with numerous possible drug and schedule regimens. We describe here how a broad panel of drug-resistant small-cell lung cancer (SCLC) cell lines can be used as a model of tumour heterogeneity to aid in the selection of non-cross-resistant regimens. We have selected low-fold (3-10x) drug-resistant sublines from a classic (NCI-H69) and a variant (OC-NYH) SCLC cell line. The resistant cell lines include two sublines with different phenotypes towards alkylating agents (H69/BCNU and NYH/CIS), two sublines with different phenotypes against topo I poisons (NYH/CAM and NYH/TPT) and three multidrug resistant (MDR) sublines (H69/DAU, NYH/VM, and H69/VP) with combinations of mdr1 and MRP overexpression as well as topoisomerase II (topo II) down-regulation or mutation. Sensitivity to 20 established and new agents was measured in a standardized clonogenic assay. Resistance was highly drug specific. Thus, none of the cell lines was resistant to all drugs. In fact, all resistant cell lines exhibited patterns of collateral sensitivity to various different classes of drugs. The most intriguing pattern was collateral sensitivity to gemcitabin in two cell lines and to ara-C in five drug-resistant cell lines, i.e. in all lines except the lines resistant to topo I poisons. Next, all sensitivity patterns in the nine cell lines were compared by correlation analysis. A high correlation coefficient (CC) for a given pair of compounds indicates a similar pattern in response in the set of cell lines. Such data corroborate the view that there is cross-resistance among the drugs. A numerically low coefficient indicates that the two drugs are acting in different ways, suggesting a lack of cross-resistance between the drugs, and a negative correlation coefficient implies that two drugs exhibit collateral sensitivity. The most negative CCs (%) to the new drug leads were: taxotere-carmustine (BCNU) (-75), taxol-cisplatin (-58), ara-C-taxol (-25), gemcitabin-doxorubicin (-32), camptotecin-VM26 (-41) and topotecan-VP16 (-17). The most negative correlations to the clinically important agent VP-16 were: cisplatin (-70); BCNU (-68); camptothecin (-38); bleomycin (-33), gemcitabin (-32); ara-C (-21); topotecan (-17); melphalan (-3); and to the other main drug in SCLC treatment cisplatin were: doxorubicin (-70); VP-16 (-70); VM-26 (-69); mAMSA (-64); taxotere (-58); taxol (-58). Taxol and taxotere were highly correlated (cross-resistant) to VP-16 (0.76 and 0.81 respectively) and inversely correlated to cisplatin (both -0.58). Similarly, camptothecin and topotecan were correlated to cisplatin but inversely correlated to VP-16 and other topo II poisons. From the sensitivity data, we conclude that collateral sensitivity and lack of cross-resistance favours a cisplatin-taxane or topo I-topo II poison combination, whereas patterns of cross-resistance suggest that epipodophyllotoxin-taxane or topo I poison-cisplatin combinations may be disadvantageous.


British Journal of Cancer (1997) 75(6), 869–877
© 1997 Cancer Research Campaign

### In vitro cross-resistance and collateral sensitivity in seven resistant small-cell lung cancer cell lines: preclinical identification of suitable drug partners to taxotere, taxol, topotecan and gemcitabin

**PB Jensen[1], B Holm[1], M Sorensen[1], IJ Christensen[2] and M Sehested[3]**

[1]Laboratory of Experimental Medical Oncology, The Finsen Center, Rigshospitalet, 9 Blegdamsvej, DK-2100 Copenhagen, Denmark; [2]The Finsen Laboratory, Rigshospitalet, DK-2100 Copenhagen; [3]Department of Pathology, Righospitalet, DK-2100 Copenhagen

**Summary** The acquisition of drug-resistant tumour cells is the main problem in the medical treatment of a range of malignant diseases. In recent years, three new classes of anti-cancer agents, each with a novel mechanism of action, have been brought forward to clinical trials. These are the topoisomerase I (topo I) poisons topotecan and irinotecan, which are both camptothecin derivatives, the taxane tubulin stabilizers taxol and taxotere and, finally, the antimetabolite gemcitabin, which is active in solid tumours. The process of optimizing their use in a combination with established agents is very complex, with numerous possible drug and schedule regimens. We describe here how a broad panel of drug-resistant small-cell lung cancer (SCLC) cell lines can be used as a model of tumour heterogeneity to aid in the selection of non-cross-resistant regimens. We have selected low-fold (3–10×) drug-resistant sublines from a classic (NCI-H69) and a variant (OC-NYH) SCLC cell line. The resistant cell lines include two sublines with different phenotypes towards alkylating agents (H69/BCNU and NYH/CIS), two sublines with different phenotypes against topo I poisons (NYH/CAM and NYH/TPT) and three multidrug resistant (MDR) sublines (H69/DAU, NYH/VM, and H69/VP) with combinations of mdr1 and MRP overexpression as well as topoisomerase II (topo II) down-regulation or mutation. Sensitivity to 20 established and new agents was measured in a standardized clonogenic assay. Resistance was highly drug specific. Thus, none of the cell lines was resistant to all drugs. In fact, all resistant cell lines exhibited patterns of collateral sensitivity to various different classes of drugs. The most intriguing pattern was collateral sensitivity to gemcitabin in two cell lines and to ara-C in five drug-resistant cell lines, i.e. in all lines except the lines resistant to topo I poisons. Next, all sensitivity patterns in the nine cell lines were compared by correlation analysis. A high correlation coefficient (CC) for a given pair of compounds indicates a similar pattern in response in the set of cell lines. Such data corroborate the view that there is cross-resistance among the drugs. A numerically low coefficient indicates that the two drugs are acting in different ways, suggesting a lack of cross-resistance between the drugs, and a negative correlation coefficient implies that two drugs exhibit collateral sensitivity. The most negative CCs (%) to the new drug leads were: taxotere–carmustine (BCNU) (–75), taxol–cisplatin (–58), ara-C–taxol (–25), gemcitabin–doxorubicin (–32), camptotecin–VM26 (–41) and topotecan–VP16 (–17). The most negative correlations to the clinically important agent VP-16 were: cisplatin (–70); BCNU (–68); camptothecin (–38); bleomycin (–33), gemcitabin (–32); ara-C (–21); topotecan (–17); melphalan (–3); and to the other main drug in SCLC treatment cisplatin were: doxorubicin (–70); VP-16 (–70); VM-26 (–69); mAMSA (–64); taxotere (–58); taxol (–58). Taxol and taxotere were highly correlated (cross-resistant) to VP-16 (0.76 and 0.81 respectively) and inversely correlated to cisplatin (both –0.58). Similarly, camptothecin and topotecan were correlated to cisplatin but inversely correlated to VP-16 and other topo II poisons. From the sensitivity data, we conclude that collateral sensitivity and lack of cross-resistance favours a cisplatin–taxane or topo I–topo II poison combination, whereas patterns of cross-resistance suggest that epipodophyllotoxin–taxane or topo I poison–cisplatin combinations may be disadvantageous.

**Keywords:** clonogenic assay; multidrug resistance; resistance to alkylating agents and topoisomerase I poisons; collateral sensitivity; new drug combinations

The treatment of small-cell lung cancer (SCLC) is currently undertaken by a few drug types, which include alkylating agents such as cisplatin and cyclophosphamide, topoisomerase II (topo II) poisons such as etoposide (VP-16) and doxorubicin, and tubulin-destabilizing drugs such as vincristine. The final treatment failure in the great majority of patients despite primary response rates of approximately 80% is considered to be due to the emergence of drug-resistant cell populations. The clonal evolution hypothesis of tumour development furthers the idea of using families of wild-type and resistant cells in an attempt to model the clinical situation and reflect the known tumour heterogeneity. Several investigators have studied the drug sensitivity of panels of SCLC cell lines in vitro. Some investigators have not been able to demonstrate any differences in the sensitivity patterns to different drug types with different mechanisms of action, such as etoposide (VP-16) and cisplatin, in large panels of wild type cell lines. Such data suggest that treatment failure is due to the presence of a pan-resistant phenotype (Tsai et al, 1990). This finding disagrees with the notion that resistance mechanisms are drug-type specific. Also, some investigators have described cell lines with a very high sensitivity to all drugs tested. This has led to the suggestion that the success in

*Received 8 July 1996*

*Revised 7 October 1996*

*Accepted 8 October 1996*

*Correspondence to:* PB Jensen primary treatment of SCLC is as the result of the initial presence of a multidrug-sensitive phenotype (Giaccone et al, 1992). If it is correct that the primary drug sensitivity is a result of hypersensitivity and that drug resistance is due to the loss of a programmed cell death or to the loss of other common pathways for cell death, the search for new active drugs would definitely appear to be hopeless. In contrast to these two extremes, i.e. the ultimate presence of a pan-resistant phenotype or the initial presence of a multidrug-sensitive phenotype, we and others have found differential sensitivity patterns when investigating the cytotoxicity of various compounds in panels of cell lines (Schabel et al, 1983; Jensen et al, 1992, 1993*a*; Weinstein et al, 1992; Koutsoukos et al, 1994). Accordingly, one way of circumventing current drug resistance would be to develop new drug types that can act on cellular targets other than those already in use. In recent years, three new classes of anti-cancer agents each with a novel mechanism of action have been brought forward to clinical trials. These are the topoisomerase I (topo I) poisons topotecan and irinotecan, which are both camptothecin derivatives, the taxane tubulin stabilizers taxol and taxotere and finally the antimetabolite gemcitabin, which is active in solid tumours. In order to supply knowledge as to appropriate combinations of standard drugs with these new drugs, we have performed a preclinical evaluation of drug combinations using a standardized clonogenic assay system on two wild type SCLC cell lines, NCl-H69, a classic type, and OC-NYH, which belongs to the variant type, and their drug-resistant sublines as a preclinical model of SCLC. Our results indicate that drug resistance is indeed drug specific as none of our cell lines are resistant to all drugs. From the sensitivity data we conclude that collateral sensitivity and lack of cross-resistance favours a cisplatin–taxane or topo I–topo II poison combination, whereas patterns of cross-resistance suggest that epipodophyllotoxin–taxane or topo I poison–cisplatin may be inappropriate.

Table 1 DNA content, plating efficiency, relation to chemotherapy and mechanism of resistance

| Cell line | DI | PE (%) | Prior therapy | Mechanism of resistance |
|---|---|---|---|---|
| NCI-H69 | 0.90 | 12 | Yes | |
| H69/DAU | 0.87 | 12 | | mdr1 overexpression, Topo II down-regulation |
| 69/VP | 0.82 | 13 | | MRP and mdr1 overexpression, normal topo II level but extranuclear localization |
| H69/BCNU | ND | 20 | | O6-methylguanine-DNA-methyltransferase overexpression |
| OC-NYH | 1.39 | 27 | No | |
| NYH/VM | 1.29 | 30 | | Topo II down-regulation |
| NYH/TPT | 1.10 | 27 | | Topo I down-regulation, topo II up-regulation |
| NYH/CAM | 1.16 | 31 | | No topo II change, unknown downstream change |
| NYH/CIS | 1.14 | 30 | | Glutathione overexpression |

DI, DNA index; PE, plating efficiency at approximately 3000 colonies; ND, not determined. OC-NYH and its sublines grow as monolayers and NCI-H69 and its sublines grow in suspension.

### MATERIALS AND METHODS

### Drugs

$O^6$-benzylguanine was kindly supplied by Dr Robert C Moschel, Frederick Cancer Research and Development Center, Frederick, MD, USA. $O^6$-benzylguanin was dissolved in dimethyl sulphoxide (DMSO). Melphalan (Wellcome) was dissolved in hydrochloric acid with ethanol and further diluted in propyleneglycol phosphate buffer; m-AMSA (Parke-Davis) was delivered in *N,N*-dimethylacetamid solution and further diluted in acid lactose; and ara-C (cytosine arabinoside) (Upjohn) was dissolved in benzyl alcohol. All the solvents used were dispensed by the producers. Doxorubicin (Farmitalia Carlo Erba Pharmacia), bleomycin (Lundbeck), hydroxyurea (Bristol-Myers Squibb), mitomycin C (Kyowa), gemcitabin (Lilly), vincristine (Lilly) and topotecan (SmithKline Beecham) were dissolved in sterile water. Vindesine (Lilly) was dissolved in isotonic sodium chloride. Camptothecin (Sigma), taxotere (Rhone-Poulenc Rohrer) and taxol (Bristol-Myers Squibb) were dissolved in DMSO. BCNU (carmustine) (Bristol-Myers Squibb) was dissolved in 10% (v/v) ethanol in sterile water. Mitoxantrone (Lederle), VP-16 (etoposide) (Bristol-Myers Squibb), VM-26 (teniposide) (Bristol-Myers Squibb) and cisplatin (Bristol-Myers Squibb) were in solution for infusion. The drugs were diluted with tissue culture medium to 300 × final concentrations, partitioned into multiple aliquots, frozen on ethanol–dry ice and stored at –80°C. Just before culture application, the contents of the frozen vials were thawed and mixed. As described in Jensen et al (1993*a*), the cytotoxic stability of the frozen drugs stored at –80°C for 30–40 days was checked by comparing with freshly diluted drug in a clonogenic assay. All drugs were checked in this setting.

### Cell lines

The human SCLC cell lines used are the classic type NCI-H69 (Carney et al, 1985) and the variant type OC-NYH (de Leij et al, 1985). The multidrug-resistant (MDR) SCLC cell lines used were H69/DAU, H69/VP and OC-NYH/VM, selected for resistance to daunorubicin, VP-16 and VM-26 respectively. H69/DAU is a classical MDR cell line with P-glycoprotein in the cell membrane (Jensen et al, 1989) and a reduced level of topo IIα; NYH/VM is resistant because of reduced topo-IIα activity and content (Jensen et al, 1993*b*); and H69/VP exhibits the multidrug resistance protein (MRP) (Brock et al, 1995), P-glycoprotein (Jensen et al, 1992) and a cytoplasmatic distribution of the target enzyme DNA topo II, presumably due to a mutation in a nuclear localization sequence (unpublished observation). The topotecan-resistant NYH/TPT cells exhibit a 50% reduction in topo I content and a doubling of the topo II level (Sorensen et al, 1995), whereas the camptothecin-selected NYH/CAM cells have an, until now, unexplained mechanism of resistance involving an unchanged topo I level and catalytic activity and a slightly increased topo II content (manuscript in preparation). NYH/CIS and H69/BCNU, selected for cisplatin and BCNU resistance, respectively, are characterized in this report. Resistant cell lines were grown in vitro without drug for a minimum of 5 days before testing. All cell lines were maintained at 37°C in RPMI 1640 with 10% fetal calf serum in a humidified atmosphere with 7.5% carbon dioxide. At regular intervals, the panel of cell lines was re-established from frozen subcultures to reduce or avoid sensitivity drifting. The cell lines

© Cancer Research Campaign 1997

**Figure 1** The relative sensitivity to 20 anti-cancer agents in the multidrug-resistant cell lines H69/DAU (**A**) and H69/VP (**B**) compared with the parental line NCI-H69 and in NYH/VM compared with the parental line OC-NYH (**C**). For a given pair of cell lines the mean $LD_{50}$ was set to 100% for each drug. The plot shows the $LD_{50}$ values of each cell line relative to mean $LD_{50}$ values. Results from at least three experiments. Bars represent two s.e.m. A star denotes the sensitivity of the resistant cell line and a circle the wild type line. If, for a given drug, a star is above a circle the resistant cell line exhibits cross-resistance to the drug; if a star is below a circle, the resistant cell line exhibits collateral sensitivity to the drug. Five different targets or mechanisms of action are denoted on the top of the figure: tubulin, topo II, topo I, alkylating and antimetabolites. Aclarubicin and bleomycin do not fit in with these five drug types. O, wild-type cell line. ✱Resistant subline

© Cancer Research Campaign 1997

**Figure 2** The relative sensitivity to 20 anti-cancer agents in the cell lines H69/BCNU and NYH/CIS selected for resistance to the alkylating agents BCNU and cisplatin. **A** compares H69/BCNU with H69 and **B** shows NYH/CIS compared with NYH. For a given pair of cell lines, the mean $LD_{50}$ was set to 100% for each drug. The plot shows the $LD_{50}$ values of each cell line relative to mean $LD_{50}$ values. Results from at least three experiments. Bars represent two s.e.m. See legend to figure 1

were free of mycoplasm contamination. DNA content (Vindeløv and Christensen, 1990), plating efficiency, relation to chemotherapy, mechanism of resistance and growth behaviour in vitro of the cell lines used are described in Table 1.

### Cellular glutathione content

Glutathione conjugation represents a major detoxification reaction in the deactivation of xenobiotics. Cells with resistance towards alkylating agents often exhibit an increased level of glutathione. DTNP (5,5′-dithio-bis(2-nitrobenzoic acid), NADPH (β-nicotinamide adenine dinucleotide phosphate), glutathione reductase, glutathione, imidazole and imidazole hydrochloride were all from Sigma. 2–4×$10^6$ cells were washed in ice-cold phosphate-buffered saline (PBS) and collected by centrifugation at 3000 r.p.m. for 3 min at 4°C. Protein was precipitated by adding 500 μl of 20% ice-cold trichloroacetic acid (TCA). This was mixed vigorously and incubated at 4°C for 15 min and extracts were neutralized to pH 7.0 by adding 400 μl of 2.1 M potassium hydroxide–1 M imidazole base–0.5 M potassium chloride and kept on ice for 15 min. The mixture was centrifugated at 10 000 r.p.m. for 2 min at 4°C. The pellet was saved for protein determination. The supernatant was analysed for total glutathione content through enzyme recycling under conditions similar to those described by Tietze (1969).

### Modulation of sensitivity with $O^6$-benzylguanine

One well-characterized mechanism of drug resistance to alkylating agents involves the DNA repair protein of $O^6$-methylguanine-DNA methyltransferase, which removes alkyl adducts from the $O^6$-position of guanine in DNA (Dolan et al, 1990). Cells in single-cell suspension were incubated for 1 hour with $O^6$-benzylguanine (20 uM) and were then exposed for 2 hours to a range of BCNU concentrations; the cells were subsequently washed in PBS × 2 and plated in the presence of 20 uM $O^6$-benzylguanine on top of a feeder layer, as explained below in the clonogenic assay section.

### Clonogenic assay

We have previously demonstrated that the comparison of effects of different drugs in a cell line is more reliable when the drugs are compared in simultaneous experiments on the same batch of cells. To obtain more dose–response curves on one batch of cells, we therefore developed an automatic colony counter (Jensen et al, 1993*a*). In each experiment, all 20 drugs (three concentrations of each, all plated in triplicate) and six control triplicates were tested on the same batch of cells. Single-cell suspensions (1–4 × $10^4$ cells $ml^{-1}$) in RPMI 1640 supplemented with 10% fetal calf serum were plated in soft agar on a feeder layer containing sheep red blood cells (Roed et al, 1987) in 35-mm Petri dishes with the desired drug concentrations (continuous incubation). The number of cells were adjusted to obtain 2000–3000 colonies in the control dishes. Solvent concentrations never exceeded 1% and had no influence on the plating efficiency. Plating was carried out within 1 h as the intraexperimental variation in plating efficiency of the controls exceeded 10% in more prolonged experiments. After 14–21 days, the colonies were counted on the image analysis system. Colonies larger than 50 μm in diameter were regarded as positive. The colony counter was interfaced with a computer and data were stored and analysed through use of SAS software. The dose reducing the number of colonies to 50% of control ($LD_{50}$) was determined from

© Cancer Research Campaign 1997

**Figure 3** Dose–response curves based on the clonogenic assay demonstrating the effect of O⁶-benzylguanine on the sensitivity to BCNU on H69 and H69/BCNU cell lines. A non-toxic dose of O⁶-benzylguanin almost completely restores sensitivity to BCNU in H69/BCNU to wild type levels (— •) and (- - -), whereas the modulator has no effect on BCNU sensitivity in the wild type line NCI-H69 (the two curves to the left). Bars represent two s.e.m. from triplicate cultures three drug concentration points in linear regression analysis on logarithmically transformed response data (Jensen et al, 1993*a*). The drug concentrations chosen approximated to $LD_{10}$, $LD_{50}$ and $LD_{90}$ obtained on cell line OC-NYH from dose–response curves in previous experiments and were as follows (μM): BCNU (0.9, 2.3, 7.0); ACLA (0.0037, 0.012, 0.025); DOX (0.026, 0.074, 0.13); MELPHAL (0.33, 0.9, 1.6); ARAC (0.025, 0.075, 0.15); BLEOMY (0.02, 0.07, 0.14); CAMPTO (0.0014, 0.0028, 0.0056); CISPT (0.33, 0.66, 1.3); HYDREA (39, 79, 237); MAMSA (0.05, 0.1, 0.3); MITO (0.014, 0.045, 0.09); MITOMY (0.009, 0.021, 0.06); VINCRI (0.001, 0.002, 0.004); VINDES (0.001, 0.002, 0.003); VM26 (0.02, 0.05, 0.1); VP16 (0.125, 0.3, 0.6); TAXOL (0.0007, 0.0021, 0.0042); TAXOTERE (0.0002, 0.0004, 0.0011); TOPOTE (0.0022, 0.0066, 0.013); GEMCIT (0.0017, 0.0033, 0.017). When the calculated $LD_{50}$ values were above three times the highest tested concentration, the $LD_{50}$ was assigned this value (i.e. $3 \times LD_{90}$ on OC-NYH). Patterns in sensitivity were studied by correlation analysis using rank orders of sensitivity with all possible pairings of the 20 agents.

At least thee experiments were included for each drug and cell line. Computations used correlation coefficients calculated as Spearman rank-order correlations.

### RESULTS

### Sensitivity patterns in resistant cell lines

Drug cytotoxicity was determined in a clonogenic assay as described in Materials and methods. The relative sensitivity of the wild-type cell line NCI-H69 compared with H69/DAU is shown in Figure 1A. H69/DAU is a multidrug-resistant cell line exhibiting P-glycoprotein. Note that there is collateral sensitivity (CS) to cisplatin as well as to ara-C and to the ara-C analogue gemcitabin. In fact, the collateral sensitivity to ara-C is eightfold. Furthermore, although there is no cross-resistance to camptothecin, there is statistically significant cross-resistance (CR) to topotecan in H69/DAU. Similar data have been published previously (Chen et al, 1991; Hendriks et al, 1992). As expected from other studies, there is also cross-resistance to taxol and taxotere in this P-glycoprotein-positive cell line.

In Figure 1B, NCI-H69 is compared with H69/VP cells which were selected for etoposide (VP-16) resistance. H69/VP exhibits the multidrug resistance protein (MRP) as well as P-glycoprotein

**Table 2** Summary of sensitivity patterns to 20 anti-cancer agents in seven drug-resistant cell lines. A blank field signifies cross-resistance, 0 signifies non-cross-resistance and CS collateral sensitivity, i.e. compared with the parental cell line, the cells have become significantly more sensitive to the drug

| | H69/DAU | H69/VP | H69/BCNU | NYH/VM | NYH/CIS | NYH/TPT | NYH/CAM |
|---|---|---|---|---|---|---|---|
| Vindesine | | | CS | 0 | 0 | | 0 |
| Vincristine | | | CS | 0 | 0 | 0 | 0 |
| Taxol | | | CS | | | 0 | 0 |
| Taxotere | | | CS | | | 0 | 0 |
| Doxorubicin | | | CS | | 0 | 0 | CS |
| Mitoxantrone | | 0 | CS | | 0 | | 0 |
| m-AMSA | | | CS | | 0 | 0 | 0 |
| VP-16 | | | CS | | 0 | 0 | CS |
| VM-26 | | | CS | | CS | CS | CS |
| Aclarubicin | | | 0 | 0 | 0 | 0 | 0 |
| Camptothecin | 0 | 0 | 0 | 0 | | | |
| Topotecan | | 0 | 0 | 0 | | | |
| Mitomycin | 0 | CS | CS | 0 | | | 0 |
| Melphalan | 0 | 0 | 0 | 0 | | | |
| BCNU | 0 | 0 | | 0 | | 0 | 0 |
| Cisplatin | CS | CS | 0 | 0 | | | |
| Bleomycin | 0 | 0 | 0 | 0 | 0 | 0 | 0 |
| Ara-C | CS | CS | CS | CS | CS | 0 | 0 |
| Gemcitabin | CS | 0 | CS | 0 | 0 | 0 | 0 |
| Hydrea | 0 | CS | 0 | 0 | | 0 | CS |

© Cancer Research Campaign 1997 

**Table 3** Correlation analysis on rank order of sensitivity with all possible pairings of the six new drug leads to the 19 other anti-cancer agents. Correlation coefficients (%) were obtained in two wild type lines and seven drug-resistant sublines. A positive correlation indicates that the sensitivity patterns overlap, i.e. the drugs are effective on the same clones (cross-resistance); a negative correlation signifies that the drugs exhibit opposite patterns (collateral sensitivity)

| | Gemcitabin | ara-C | Taxotere | Taxol | Topotecan | Camptothecin |
|---|---|---|---|---|---|---|
| Vindesine | 14 | 2 | 52 | 59 | 51 | −2 |
| Vincristine | 13 | −2 | 80 | 76 | 13 | −4 |
| Taxol | −24 | −25 | 76 | | 28 | −23 |
| Taxotere | −9 | −5 | | 76 | 21 | −33 |
| Doxorubicin | −32 | −13 | 75 | 77 | −11 | −29 |
| Mitoxantrone | 8 | 11 | 83 | 68 | 11 | −18 |
| mAMSA | −30 | −24 | 88 | 76 | 5 | −32 |
| VP-16 | −32 | −21 | 81 | 76 | −17 | −38 |
| VM-26 | −25 | −22 | 77 | 69 | −14 | −41 |
| Aclarubicin | −4 | −12 | 63 | 59 | 46 | 22 |
| Camptothecin | 38 | 26 | −33 | −23 | 69 | |
| Topotecan | 33 | 3 | 21 | 28 | | 69 |
| Mitomycin | 27 | 33 | 59 | 41 | 45 | 40 |
| Melphalan | 25 | −22 | 7 | 20 | 81 | 65 |
| BCNU | −13 | −13 | −75 | −52 | 4 | 5 |
| Cisplatin | 41 | 16 | −58 | −58 | 33 | 55 |
| Bleomycin | 29 | −3 | −35 | −14 | 6 | 8 |
| Ara-C | 60 | | −5 | −26 | 3 | 26 |
| Gemcitabin | | 60 | −9 | −24 | 33 | 38 |
| Hydrea | 10 | 35 | 43 | 9 | 32 | 33 |

**Figure 4** Sensitivity patterns to camptothecin, VP-16, cisplatin and taxol on the two wild-type and seven resistant SCLC cell lines. The results are depicted as the mean relative $LD_{50}$ values from at least three experiments and the cell lines are sorted by increasing sensitivity to cisplatin. Bars are plus one s.e.m. CIS, NYH/CIS; TPT, NYH/TPT; CAM, NYH/CAM; BCNU, H69/BCNU; H69, NCI-H69; NYH, OC-NYH; VM, NYH/VM; VP, H69/VP; DAU, H69/DAU

and, in addition, immunohistochemistry demonstrates a clear cytoplasmatic localization of the etoposide target enzyme topo-IIα (not shown). Thus, three different mechanisms of etoposide resistance are simultaneously present in this cell line. In spite of this, the etoposide resistance is not several logs but only a factor of three to four as seen in Figure 1B. H69/VP exhibits collateral sensitivity to mitomycin and hydrea and, similar to the P-glycoprotein positive H69/DAU, the subline also exhibits collateral sensitivity to cisplatin and ara-C. In contrast to H69/DAU, however, there is no cross-resistance to topotecan in this line. This could be explained by the fact that the level of P-glycoprotein in H69/VP is much lower than in H69/DAU (Brock et al, 1995).

In Figure 1C, the wild-type cell line OC-NYH is compared with NYH/VM. NYH/VM was selected for teniposide (VM-26) resistance, the cell line exhibits a two- to threefold reduced topo-II activity and content of both the α and β form (Jensen et al, 1993 *b*). Observe a slight cross-resistance to taxol and taxotere but no cross-resistance to vincristine or vindesine and no resistance to topotecan. Also, this subline exhibits collateral sensitivity to ara-C.

In Figure 2A, H69 is compared with H69/BCNU. H69/BCNU is almost exclusively resistant to BCNU with a trend towards cross-resistance to topotecan. Also, this subline exhibits a collateral sensitivity to ara-C.

In Figure 2A, H69 is compared with H69/BCNU. H69/BCNU is almost exclusively resistant to BCNU with a trend towards cross resistance to melphalan and cisplatin. Curiously, the cell line exhibits collateral sensitivity to a number of anti-cancer agents, not only to all topo II-targeting agents but also the tubulin-targeting drugs and ara-C. The cell line was studied in the presence of $O^6$-benzylguanine. As seen in Figure 3, $O^6$-benzylguanine completely restores the sensitivity to BCNU in H69/BCNU to the wild type level. For comparison, $O^6$-benzylguanine had no effect on the sensitivity to BCNU in H69 cells. It is still unresolved whether the increased $O^6$-methylguanine-DNA methyltransferase expression is involved in the pattern of multiple collateral sensitivity to other agents.

© Cancer Research Campaign 1997

In Figure 2B OC-NYH is compared with NYH/CIS. NYH/CIS exhibits cross-resistance to all alkylating agents. In addition there is cross-resistance to hydrea, to topo I poisons and slight cross-resistance to the taxanes. There is no cross-resistance to topo II poisons or ara-C–gemcitabin; in fact, there is collateral sensitivity to VM-26 and ara-C. In three experiments, glutathione levels were 1.4- to 2.4-fold (median 1.6-fold) higher in NYH/CIS than in NYH. Median glutathione levels in NYH/CIS and NYH were 0.85 and 0.5 nmol $10^{-6}$ cells respectively. Accordingly, an increased level of glutathione in NYH/CIS is one plausible mechanism of resistance to cisplatin, BCNU and melphalan.

In Table 2, the sensitivity patterns of all resistant sublines are summarized, including the cell lines selected with the topo I poisons NYH/TPT and NYH/CAM.

In Figure 4, the cell lines are ranked according to sensitivity to cisplatin. As seen at the bottom of the figure, the pattern to taxol is almost the reverse of the pattern to cisplatin, i.e. cell lines resistant to the one drug are sensitive to the other. On the top panel is shown the patterns to camptothecin and etoposide. The pattern to camptothecin resembles the pattern to cisplatin and is the reverse of etoposide.

To compare the possible drug pairings, we performed a correlation analysis using rank orders of sensitivity with all possible pairings of the 20 agents. A high correlation coefficient (CC) for a given pair of compounds indicates a similar pattern in response in the set of cell lines. Such data corroborate the view that there is cross-resistance among the drugs. A numerically low coefficient indicates that the two drugs are acting in different ways suggesting a lack of cross-resistance between the drugs, and finally a negative correlation coefficient implies that two drugs exhibit collateral sensitivity. Table 3 shows the Spearman correlation coefficients (CCs) to gemcitabin, ara-C, taxotere, taxol, topotecan and camptothecin. The most negative CCs to the new drug leads were: taxotere/BCNU (–75), taxol–cisplatin (–58), ara-c–taxol (–25), gemcitabin–doxorubicin (–32), camptothecin–VM26 (–41), topotecan–VP16 (–17).

In SCLC, the two most widely used drugs are etoposide and cisplatin, and we therefore ranked their CCs to the other 19 drugs as follows.

(a) Ranking the correlations to VP-16:

cisplatin (–70) BCNU (–68) camptothecin (–38) bleomycin (–33) gemcitabin (–32) cytosine arabinoside (–21) topotecan (–17) melphalan (–3) hydroxyurea (14) mitomycin C (26) vindesine (32) aclarubicin (50) vincristine (63) mitoxantrone (73) taxol (76) taxotere (81) m-AMSA (89) doxorubicin (92) tenoposide (97)

(b) Ranking the correlations to cisplatin:

doxorubicin (–70) etoposide (–70) teniposide (–69) m-AMSA (–64) taxotere (–58) taxol (–58) mitoxantrone (–54) vincristine (–50) aclarubicin (–44) vindesine (–35) mitomycin C (–2) hydrea (5) ARAC (16) melphalan (29) bleomycin (32) topotecan (33) gemcitabin (41) camptothecin (55) carmustine (68)

### DISCUSSION

Treatment of SCLC often includes either the combination of CAV, i.e. cyclophosphamide + doxorubicin + vincristine, or PE, i.e. cisplatin + etoposide. The latter is considered by many oncologists to be the golden standard in the treatment of SCLC today. It is notable that two of three MDR cell lines exhibit collateral sensitivity (CS) to cisplatinum and that NYH/CIS exhibits CS to teniposide (VM-26 in Table 2). This inverse correlation has been a puzzle for a long time (Tan et al, 1987). The phenomenon is even more striking when turning to the comparison of variations in sensitivity. Thus, in this study, the sensitivity pattern to cisplatin is inversely correlated to the patterns of etoposide and teniposide (correlation coefficients –69% and –70% respectively) (Figure 4). It has been suggested that DNA topo II is involved in DNA repair; accordingly, a low topo II content, which would convey resistance to topo II poisons, would diminish DNA repair capacity and lead to hypersensitivity to cisplatin and vice versa. This hypothesis was recently tested in a cell line transfected to overexpress topo II and, indeed, this line exhibited increased sensitivity to topo II poisons and decreased sensitivity to cisplatin (Eder et al, 1995). CAV or PE regimens give remissions in 80% of patients but are obviously seldom sufficient to cure the patients, and new drugs with activity in the doubly resistant cell populations are urgently needed. Thus, the identification of drugs with effect in the etoposide and cisplatin-resistant phenotype appears to be particulary important. In recent years, three new classes of anti-cancer agents each with a novel mechanism of action have been brought forward to clinical trials. These are the antimetabolite gemcitabin, which is active in solid tumors, the topo I poisons topotecan and irinotecan, which are both camptothecin derivatives and, finally, the taxane tubulin stabilizers taxol and taxotere.

### Gemcitabin and ara-C

The sensitivity pattern to ara-C is inversely correlated for the topo II poisons doxorubicin (CC – 12) and etoposide (CC–21). In addition, all the MDR cell lines that are cross-resistant to topo II poisons exhibit collateral sensitivity to ara-C. Thus, the MDR cells have become more sensitive to ara-C than their parental wild type cells. This clearly suggests that it might be of benefit to combine a topo II poison and ara-C. Interestingly, the 3+7 combination of the topo II poison daunorubicin and ara-C is very important in the treatment of acute myeloblastic leukaemia (Keating et al, 1993). Unfortunately, ara-C is not clinically active in SCLC but the ara-C analogue, gemcitabin, has demonstrated response rates of 20% in non-small-cell lung cancer (Abratt et al, 1994; Anderson et al, 1994) and 27% in SCLC (Cormier et al, 1994). However, it is unfortunate that gemcitabin does not exhibit a sensitivity pattern identical to that of ara-C. As seen in Table 2, five of the resistant sublines exhibit collateral sensitivity (CS) to ara-C whereas only two of the lines exhibit CS to gemcitabin. In accordance with this, the ara-C–gemcitabin correlation of only 60% also indicates some difference between their mechanisms of action or their cellular pharmacokinetics. As seen in Table 2, the topo I-resistant lines NYH/TPT and NYH/CAM have unaltered sensitivity to ara-C whereas the three MDR and the two alkylating resistant lines exhibit CS. This suggests that a compound with activity in solid tumours and with ara-C characteristics would be an extremely interesting adjunct to the classic cisplatin–etoposide or cyclophosphamide–doxorubicin–vincristine SCLC treatment protocols.

### Topo I poisons

There is a remarkable cross-resistance to camptothecin and topotecan in NYH/CIS, suggesting that increased glutathione levels may also lead to resistance to topo I poisons. Other explanations such as altered topoisomerase I activity may be more plausible, and we are currently measuring topoisomerase levels and

© Cancer Research Campaign 1997

activity in NYH/CIS. Similarly NYH/CAM and NYH/TPT exhibit cross-resistance to cisplatin. From a clinical point of view, it is worrying that resistance to camptothecin and topotecan may be linked to cisplatin resistance; thus, topo I-directed drugs may not be an independent adjunct to the standard cisplatin and etoposide regimens in SCLC.

Several observations indicate that cellular resistance to topo I-targeting drugs is associated with a decrease in enzymatic activity caused by down-regulation and/or mutation of the topo-I gene (Andoh et al, 1987; Kjeldsen et al, 1988; Sugimoto et al, 1990*a*; Tanizawa and Pommier, 1992; Sorensen et al, 1995) Cells that are resistant to camptothecin appear to depend to a greater extent than wild-type cells upon topo II activity (Sugimoto et al, 1990*b*; Oguro et al, 1990). This, in turn, can lead to collateral sensitivity to topo II-targeting agents (Sugimoto et al, 1990*b*). Thus, cells resistant to topo I poisons are, in some cases at least, hypersensitive to topo II poisons. In the present investigation, both NYH/CAM and NYH/TPT exhibit collateral sensitivity to teniposide and NYH/CAM also to etoposide and doxorubicin. Furthermore, resistance towards topo- II poisons is frequently associated with increased topo I level and/or sensitivity to camptothecin (Tan et al, 1989; Minato et al, 1990; Lefevre et al, 1991). We found no cross-resistance to camptothecin in the MDR lines. Indeed, in all three MDR lines, there is a trend towards collateral sensitivity to camptothecin. Thus, studies on cell lines resistant to topo I or II poisons have demonstrated a pattern of collateral sensitivity between these two drug types, suggesting that a sequential administration of these drugs would be beneficial. These results have made us initiate a phase II clinical trial with a schedule of sequential administration of topo I poison–platinum and a topo II poison–platinum regimen in previously untreated SCLC patients.

### Taxane plus platinum

The combination of a taxane and a platinum derivative has demonstrated high activity in a number of tumours, e.g. the well known high activity of cisplatin plus taxol in the treatment of ovarian carcinomas (McGuire et al, 1995). Also, this combination appears very active in non-SCLC (Belani et al, 1995), and there are results suggesting impressive activity in breast cancer (Gelmon, 1995). It is therefore interesting that the comparison of patterns on our panel of cell lines demonstrate inverse correlations between these drug types (Figure 4 bottom). Thus, the correlation coefficients of cisplatin–taxol and cisplatin–taxotere are both as low as –58%, i.e. an inverse pattern similar to the pattern of epipodophyllotoxin plus platinum. Therefore, the combination of a taxane and platinum is very promising because of their lack of mutual cross-resistance. In addition, the combination of a taxane and the alkylating agent BCNU appears very promising. BCNU–taxol and BCNU–taxotere show CCs of –52% and –75% respectively. These figures compare favourably with the etoposide–cisplatin correlation (–70%) and could give support for a clinical trial.

In conclusion, the differential sensitivity patterns demonstrated herein clearly support the notion that there is no cell line that alone could represent the drug-resistant phenotype. In fact, all cell lines exhibited patterns of collateral sensitivity to various different classes of drugs. The analysis of the differential cytotoxicity patterns and of patterns of collateral sensitivity enable combinations of non-cross-resistant drugs and makes it possible to obtain information about drug mechanism of action. This observation agrees with results from the National Cancer Institute (NCI) in vitro anti-tumour drug screen which showed that sensitivity data in a panel of diverse cell lines can be used to predict drug mechanism of action (Weinstein et al, 1992, Koutsoukos et al, 1994). Clearly, none of the data above may be applied clinically without caution and concern for the recognized gap between simple preclinical models and the complicated clinical reality. But, although simplified, the model does provide information that we can use and test in the design of new treatment protocols.

### ACKNOWLEDGEMENTS

Annette Nielsen and Dorotheia DaSilva are thanked for excellent technical assistance. This study was supported by grants from the Danish Cancer Society.

### ABBREVIATIONS

ACLA, aclarubicin (aclacinomycin A); DOX, doxorubicin; ARAC, cytosine arabinoside (cytarabine); BCNU, carmustine; BLEOMY, bleomycin; CISPT, cisplatin (diamminedichloroplatinum); CAMPTO, camptothecin; GEMCIT, gemcitabin; HYDREA, hydroxyurea; NSCLC, non-small-cell lung cancer; MDR, multidrug resistance; MITO, mitoxantrone; MELPHAL, melphalan; MITOMY, mitomycin C; SCLC, small-cell lung cancer; TOPO, topoisomerase; TOPOTE, topotecan; VINCRI, vincristine; VINDES, vindesine; VP-16, etoposide; VM-26, teniposide

### REFERENCES

Abratt RP, Bezwoda WR, Falkson G, Goedhals L, Hacking D and Rugg TA (1994) Efficacy and safety profile of gemcitabine in non-small-cell lung cancer: a phase II study. *J Clin Oncol* **12**: 1535–1540

Anderson H, Lund B, Bach F, Thatcher N, Walling J and Hansen HH (1994) Single-agent activity of weekly gemcitabine in advanced non-small-cell lung cancer: a phase II study. *J Clin Oncol* **12**: 1821–1826

Andoh T, Ishii K, Suzuki Y, Ikegami Y, Kusunoki Y, Takemoto Y and Okada K (1987) Characterization of a mammalian mutant with a camptothecin-resistant DNA topoisomerase I. *Proc Natl Acad Sci USA* **84**: 5565–5569

Belani CP, Aisner J, Hiponia D and Engstrom C (1995) Paclitaxel and Carboplatin with and without Filgrastrim Support in Patients with Metastatic Non-Small-Cell-Lung Cancer. *Semin Oncol* **22**: (suppl. 9): 7–12

Brock I, Hipfner DR, Nielsen BS, Jensen PB, Deeley RG, Cole SPC and Sehested M (1995) Sequential co-expression of the multidrug resistance genes, MRP and mdr1 and their products in VP-16 (etoposide) selected H69 small cell lung cancer cells. *Cancer Res* **55**: 459–462

Carney DN, Gazdar AF, Bepler G, Guccion JG, Marangos PJ, Moody TW, Zweig MH and Minna JD (1985) Establishment and identification of small cell lung cancer cell lines having classic and variant features. *Cancer Res* **45**: 2913–2923

Chen AY, YU C, Potmesil M, Wall ME, Wani MC and Liu LF (1991) Camptothecin overcomes MDR1-mediated resistance in human KB carcinoma cells. *Cancer Res* **51**: 6039–6044

Cormier Y, Eisenhaueer E, Muldal A, Gregg R, Ayoub J, Goss G, Stewart D, Tarasoff P and Wong D (1994) Gemcitabine is an active new agent in previously untreated extensive small cell lung cancer (SCLC). *Ann Oncol* **5**: 283–285

De Leij L, Postmus PE, Buys CHCM, Elema JD, Ramaekers F, Poppema S, Brouwer M, Van der Veen AY, Mesander G and The Th (1985) Characterization of three new variant type cell lines derived from small cell carcinoma of the lung. *Cancer Res* **45**: 6024–6033

Dolan ME, Moschel RC and Pegg AE (1990) Depletion of mammalian $O^6$-alkylguanine-DNA alkyltransferase activity by $O^6$-benzylguanine provides a means to evaluate the role of this protein in protection against carcinogenic and therapeutic alkylating agents. *Proc Natl Acad Sci USA* **87**: 5368–5372

Eder JP, Chan V T-W, NG S-W, Rizvi NA, Zacharoulis S, Teicher BA and Schnipper LE (1995) DNA topoisomerase II alpha expression is associated with alkylating agent resistance. *Cancer Res* **55**: 6109–6116

© Cancer Research Campaign 1997

Gelmon K (1995) Biweekly Paclitaxel in the Treatment of Patients with Metastatic Breast Cancer. *Semin Oncol* **22**: (suppl. 12) 117–122

Giaccone G, Gazdar AF, Beck H, Zunino F and Capranico G (1992) Multidrug sensitivity phenotype of human lung cancer cells associated with topoisomerase II expression. *Cancer Res* **52**: 1666–1674

Hansen HH (1992) Management of small-cell cancer of the lung. *Lancet* **339**: 846–849

Hendriks CB, Rowinsky EK, Grochow LB, Donehower RC and Kaufmann SH (1992) Effect of P-glycoprotein expression on the accumulation and cytotoxicity of topotecan (SKANDF 104864), a new camptothecin analogue. *Cancer Res* **52**: 2268–2278

Jensen PB, Vindeløv L, Roed H, Demant EJF, Sehested M, Skovsgaard T and Hansen HH (1989) In vitro evaluation of the potential of aclarubicin in the treatment of small cell carcinoma of the lung (SCCL). *Br J Cancer* **60**: 838–844

Jensen PB, Roed H, Sehested M, Demant EJF, Vindeløv L, Christensen IJ and Hansen HH (1992) Doxorubicin sensitivity pattern in a panel of small cell lung cancer cell lines: correlation to etoposide and vincristine and inverse correlation to carmustine sensitivity. *Cancer Chemother Pharmacol* **31**: 46–52

Jensen PB, Christensen IJ, Sehested M, Hansen HH and Vindeløv L (1993*a*) Differential cytotoxicity of 19 anticancer agents in wild type and etoposide resistant small cell lung cancer cell lines. *Br J Cancer* **67**: 311–320

Jensen PB, Sørensen BS, Sehested M, Demant EJF, Kjeldsen E, Friche E and Hansen HH (1993*b*) Different modes of anthracycline interaction with topoisomerase II: separate structures critical for DNA-cleavage, and for overcoming topoisomerase II-related drug resistance. *Biochem Pharmacol* **45**: 2025–2035

Keating MJ, Estey E and Kantarjian H (1993) Acute Leukemia. In *Cancer Principles and Practice of Oncology* 4th ed, De Vita VT, Hellman S and Rosenberg SA (eds), pp. 1938–1964. Lippincott: Philadelphia

Kjeldsen E, Bonven BJ, Andoh T, Ishii K, Okada K, Bolund L and Westergaard O (1988) Characterization of a camptothecin-resistant human DNA topoisomerase I. *J Biol Chem* **263**: 3912–3916

Koutsoukos AD, Rubinstein LV, Faraggi D, Simon RM, Kalyandrug S, Weinstein JN, Kohn KW and Paull KD (1994) Discrimination techniques applied to the NCI in vitro anti-tumour drug screen - predicting biochemical mechanism of action. *Statist Med* **13**: 719–730

Lefevre D, Riou JF, Ahomadegbe JC, Zhou DY, Benard J and Riou G (1991) Study of molecular markers of resistance to m-AMSA in a human breast cancer cell line. Decrease of topoisomerase II and increase of both topoisomerase I and acidic glutathione S transferase. *Biochem Pharmacol* **41**: 1967–1979

McGuire WP, Hoskins WJ, Brady MF, Kucera PR, Partridge EE, Look KY, Clarke-Pearson DL and Davidson M (1996) Cyclophosphamide and cisplatin compared with paclitaxel and cisplatin in patients with stage III and stage IV ovarian cancer. *N Engl J Med* **334**: 1–6

Minato K, Kanzawa F, Nishio K, Nakagawa K, Fujiwara Y and Saijo N (1990) Characterization of an etoposide-resistant human small-cell lung cancer cell line. *Cancer Chemother Pharmacol* **26**: 313–317

Oguro M, Seki Y, Okada K and Andoh T (1990) Collateral drug sensitivity induced in CPT-11 (a novel derivative of camptothecin)-resistant cell lines. *Biomed Pharmacother* **44**: 209–216

Roed H, Christensen IJ, Vindeløv L, Spang-Thomsen M and Hansen HH (1987) Interexperiment variation and dependence on culture conditions in assaying chemosensitivity of human small cell lung cancer lines. *Eur J Cancer Clin Oncol* **23**: 177–186

Schabel FM JR, Skipper HE, Trader MW, Laster WR Jr, Griswold DP Jr and Corbett TH (1983) Establishment of cross-resistance profiles for new agents. *Cancer Treat Rep* **67**: 905–922

Sehested M, Friche E, Jensen PB and Demant EJF (1992) Relationship of VP-16 to the classical multidrug resistance (MDR) phenotype. *Cancer Res* **52**: 2874–2879

Sorensen M, Sehested M and Jensen PB (1995) Characterisation of a human small-cell lung cancer cell line resistant to the DNA topoisomerase I-directed drug topotecan. *Br J Cancer* **72**: 399–404

Sugimoto Y, Tsukahara S, Oh Hara T, Isoe T and Tsuruo T (1990*a*) Decreased expression of DNA topoisomerase I in camptothecin-resistant tumor cell lines as determined by a monoclonal antibody. *Cancer Res* **50**: 6925–6930

Sugimoto Y, Tsukahara S, OH Hara T, Liu LF and Tsuruo T (1990*b*) Elevated expression of DNA topoisomerase II in camptothecin-resistant human tumor cell lines. *Cancer Res* **50**: 7962–7965

Tan KB, Mattern MR, Boyce RA and Schein PS (1987) Elevated DNA topoisomerase II activity in nitrogen mustard-resistant human cells. *Proc Natl Acad Sci USA* **84**: 7668–7671

Tan KB, Mattern MR, Eng W-K, McCabe FL and Johnson RK (1989) Nonproductive rearrangement of DNA topoisomerase I and II genes: correlation with resistance to topoisomerase inhibitors. *J Natl Cancer Inst* **81**: 1732–1735

Tanizawa A and Pommier Y (1992) Topoisomerase I alteration in a camptothecin-resistant cell line derived from Chinese hamster DC3F cells in culture. *Cancer Res* **52**: 1848–1854

Tietze F (1969) Enzymic method for quantitative determination of nanogram amounts of total and oxidized glutathione: applications to mammalian blood and other tissues. *Anal Biochem* **27**: 502–522

Tsai C, Ihde DC, Kadoyama C, Venzon D and Gazdar AF (1990) Correlation of in vitro drug sensitivity testing of long-term small cell lung cancer cell lines with response and survival. *Eur J Cancer* **26**: 1148–1152

Weinstein JN, Kohn KW, Grever MR, Viswanadhan VN, Rubinstein LV, Monks AP, Scudiero DA, Welch L, Koutsoukos AD, Chiausa AJ and Paull KD (1992) Neural computing in cancer drug development: predicting mechanism of action. *Science* **258**: 447–451

Vindeløv L and Christensen IJ (1990) A review of techniques and results obtained in one laboratory by an integrated system of methods designed for routine flow cytometric DNA analysis. *Cytometry* **11**: 753–770

© Cancer Research Campaign 1997